# Association between preoperative controlling nutritional status score and surgical site infection following instrumented posterior lumbar interbody fusion

**DOI:** 10.3389/fsurg.2026.1918332

**Published:** 2026-09-24

**Authors:** Yongjun Liu, Xiaoyan Chen, Leisheng Wang, Yan Ding

**Affiliations:** The Second Department of Spine Surgery, Yantaishan Hospital, Yantai, Shandong, China

**Keywords:** controlling nutritional status score, nutritional status, posterior lumbar interbody fusion, spine surgery, surgical site infection

## Abstract

**Objective:**

This study aimed to investigate the association between preoperative Controlling Nutritional Status (CONUT) score and surgical site infection (SSI) after instrumented posterior lumbar interbody fusion (PLIF).

**Methods:**

This retrospective cohort study included patients who underwent instrumented PLIF for lumbar degenerative disease at our institution between January 2021 and March 2025. SSI events within 12 months postoperatively were identified through medical records and follow-up assessment. Restricted cubic spline (RCS) analysis was performed to evaluate the potential nonlinear association between CONUT score and SSI risk. Based on the observed risk pattern and clinical interpretability, an exploratory cutoff of 3 was used to dichotomize patients for categorical analysis. Multivariable logistic regression was performed to assess the independent association between CONUT score and SSI, with sensitivity analysis treating CONUT as a continuous variable.

**Results:**

Among 1,414 patients included, 43 (3.0%) developed SSI within 12 months postoperatively. RCS analysis suggested a significant nonlinear association between CONUT score and SSI risk (*P* for nonlinearity = 0.015), with risk beginning to increase at approximately a CONUT score of 3. Patients were dichotomized into CONUT < 3 and ≥3 groups. After adjustment for potential confounders, CONUT ≥3 was independently associated with an increased risk of SSI (OR, 2.49; 95% CI, 1.32–4.70; *P* = 0.005). Sensitivity analysis showed that each 1-point increase in CONUT score was associated with a 28% increase in SSI risk (OR, 1.28; 95% CI, 1.12–1.45; *P* < 0.001).

**Conclusion:**

Higher preoperative CONUT score was independently associated with increased SSI risk after instrumented PLIF. Given its accessibility and low cost, CONUT may serve as a practical indicator for preoperative nutritional assessment and SSI risk stratification, although further external validation is warranted.

## Introduction

Posterior lumbar interbody fusion (PLIF) is widely used for degenerative lumbar spinal disorders and can provide effective neural decompression and spinal stabilization. However, it is associated with a substantial number of postoperative complications, with a reported overall complication rate of up to 37.5% (1), which is nearly double that observed in transforaminal lumbar interbody fusion (TLIF) procedures (2). Among them, surgical site infection (SSI) remains a clinically important concern, prolonging hospitalization by an average of 6.9–11 days, increasing 30-day and 90-day readmission risk (7.4% and 18.5%, respectively), raising healthcare costs and compromising postoperative recovery (3–5). Given the increasing use of lumbar fusion procedures worldwide, identifying modifiable factors that can be assessed and optimized before surgery is essential for improving perioperative outcomes.

Malnutrition has increasingly been recognized as a modifiable factor associated with postoperative complications, including SSI, across several orthopaedic subspecialties (6–9). Perioperative nutritional optimization has also been reported to improve postoperative recovery in orthopaedic and spine surgery, supporting the clinical relevance of preoperative nutritional assessment (10–12). For example, the European Society for Clinical Nutrition and Metabolism (ESPEN) guidelines recommend early nutritional supplementation for all geriatric patients undergoing orthopedic procedures (13). However, the role of nutritional status in spinal surgery remains insufficiently clarified, and previous studies have reported inconsistent findings (4, 14–17). This inconsistency may partly reflect the reliance on single biomarkers, such as serum albumin or prealbumin, which can be influenced by inflammation, hepatic function, hydration status, and other perioperative conditions (18).

The Controlling Nutritional Status (CONUT) score is an integrated nutritional assessment tool based on serum albumin concentration, peripheral lymphocyte count, and total cholesterol concentration. Compared with single laboratory markers, CONUT may provide a more comprehensive reflection of nutritional reserve, immune status, and systemic physiological vulnerability, and has shown prognostic value for postoperative adverse outcomes in orthopaedic patients (19–21). Nevertheless, evidence regarding the association between CONUT score and SSI after instrumented PLIF remains limited, and the potential dose-response relationship between CONUT score and SSI risk has not been well characterized.

Therefore, the present study aimed to investigate the association between preoperative CONUT score and SSI after instrumented PLIF. We further used restricted cubic spline analysis to explore the dose-response relationship between CONUT score and SSI risk, with the goal of evaluating whether CONUT could serve as a practical indicator for preoperative nutritional risk assessment and SSI risk stratification in this population.

## Methods

This retrospective single-center study was performed in accordance with the Declaration of Helsinki. Prior to commencement, the study protocol was reviewed and approved by the Ethics Committee of the Yantaishan Hospital (**NO 2025008**), which waived the requirement for patient informed consent, as the data collection and analysis were conducted using de-identified data that ensured the anonymity of participants. Clinical trial number: Not applicable.

Patients eligible for inclusion were those aged ≥ 18 years who underwent an instrumented PLIF procedure for the treatment of a lumbar degenerative disease, that is, herniated disc disease, degenerative disc disease, spondylolisthesis, spinal stenosis, spinal instability, or a combination, in Yantaishan Hospital between January 2021 and March 2025. The exclusion criteria were disorders other than degenerative disease (trauma or tumor); operative procedures other than instrumented PLIF; pre-existing confirmed or suspected infections within 3 months before admission; pre-existing conditions or disorders affecting the serum albumin level, lymphocyte count, or TC (autoimmune hepatitis, rheumatoid arthritis, tumor), revision surgery; and incomplete follow-up data regarding SSI.

The patients were operated upon by a spinal surgery team, including three chief physicians, three deputy chief physicians, five attending physicians, and one resident physician. In accordance with standard guidelines, a single preoperative dose of prophylactic antibiotics (typically cefazolin, unless the patient had an allergy) was intravenously administered 30 min prior to skin incision. For procedures lasting > 3 h, an additional dose was administered. Postoperatively, antibiotics were typically discontinued within 24 h. For complex cases judged to be at high risk of infection, surgeons could elect to extend the duration of antibiotic prophylaxis, although this rarely exceeded three days. Specific surgical techniques for decompression, fixation, bone grafting, and drainage tube utilization were determined according to surgeon preference.

## Definition and confirmation of SSI

SSI was defined according to CDC/NHSN criteria (22). In this study, the surveillance window was extended to 12 months postoperatively to capture delayed infections after instrumented fusion. Superficial SSI refers to an infection limited to the skin and subcutaneous tissue, with possible presentations of redness, tenderness, warmth, and pain at the wound site, and can typically be resolved by local wound care and oral antibiotic treatment. Deep SSI involve deeper tissues, such as the fascia, muscle, or vertebral space, and present with severe symptoms or signs, including fever, persistent wound drainage or dehiscence, abscess formation, and gangrene, frequently necessitating additional surgical treatment. Wound culture results were considered the reference standard for diagnosis, but were not the sole determinant.

Potential SSI events were identified through multiple sources, including inpatient medical records, documented diagnoses, daily wound site descriptions during the postoperative hospitalization period, telephone follow-up at 1, 3, 6, and 12 months after discharge, and readmission records within 12 months postoperatively. All potential SSI events were independently adjudicated by two investigators according to CDC/NHSN criteria. During outcome adjudication, the investigators were blinded to the patients' CONUT scores, CONUT categories, and the individual laboratory components used to calculate CONUT, including serum albumin concentration, peripheral blood lymphocyte count, and total cholesterol concentration. Any disagreement was resolved through discussion with a senior spine surgeon.

## CONUT score calculation and evaluation

The CONUT score was an objective nutritional screening index calculated from three routinely available laboratory parameters: peripheral blood lymphocyte count, serum albumin concentration, and TC concentration. These three components reflect protein reserve, immune status, and lipid/metabolic reserve, respectively. The CONUT score ranges from 0 to 12, with higher scores indicating more severe nutritional impairment. According to the conventional CONUT classification, scores of 0–1 indicate normal nutritional status, 2–4 mild undernutrition, 5–8 moderate undernutrition, and 9–12 severe undernutrition. Details about calculation are presented in Supplementary Table 1.

Peripheral blood lymphocyte count, serum albumin concentration, and total cholesterol concentration were obtained from routine preoperative blood and biochemical examinations performed during the index hospitalization before surgery. When multiple preoperative measurements were available, the values closest to the date of surgery were used for CONUT calculation. These parameters were preferentially selected from laboratory tests performed on the same preoperative sampling date. If same-day results were unavailable, the closest available preoperative values were used. Because lymphocyte count was obtained from routine blood testing, whereas serum albumin and total cholesterol were obtained from biochemical testing, the three components were not necessarily derived from the same specimen tube. All measurements were performed in the hospital central laboratory using standardized automated analyzers according to the manufacturers' protocols.

## Covariates

Variables that might potentially influence the SSI risk or CONUT score were extracted from the medical records of the index hospitalization stay by two authors (Y.L. and X.C.). These included demographics (age, sex, residence), comorbidities (body mass index (BMI), hypertension, diabetes mellitus, heart disease, cerebrovascular disease, chronic obstructive pulmonary disease, renal disease, liver disease, peripheral vascular disease (PVD), glucocorticoid use within 3 months prior to admission), smoking status (never, former, current), operative variables (preoperative waiting in days, American Society of Anesthesiologists (ASA) classification, number of operated levels, allogeneic bone or bone substitute, type of preoperative antibiotic use, intraoperative blood loss, allogeneic blood transfusion, and surgical duration in minutes).

## Statistical analysis

Continuous variables were summarized using mean and standard deviation (SD), and normality was assessed using the Kolmogorov–Smirnov test. Depending on the normality status, either Student's t-test or Mann–Whitney U test was used for between-group comparisons, as appropriate. Categorical data are presented as counts and percentages, with between-group differences detected using the *Chi-square* test or *Fisher's exact* test, as appropriate.

Restricted cubic spline (RCS) analysis was performed to explore the potential dose-response and nonlinear relationship between the preoperative CONUT score and the risk of SSI. A logistic regression model incorporating RCS terms for the CONUT score was fitted, with a CONUT score of 0 used as the reference value. Considering the discrete distribution of the CONUT score in the present cohort, internal knots were placed at CONUT scores of 2 and 5, corresponding to the transitions from normal to mild and from mild to moderate undernutrition, respectively; An additional knot at the transition to severe undernutrition (CONUT score = 9) was not used because the number of patients with severe undernutrition was very small, which could have led to unstable estimation. Boundary knots were set at the minimum and maximum observed values. The odds ratios (ORs) and 95% confidence intervals (CIs) derived from the RCS model were plotted to visualize the association between CONUT score and SSI risk. The overall association was assessed using a likelihood ratio test comparing the RCS model with the null model, and nonlinearity was evaluated by comparing the RCS model with a model containing CONUT as a linear term.

For clinical interpretability, and as an exploratory analysis based on the risk pattern observed in the RCS curve, the CONUT score was categorized using a cutoff of 3. This threshold was not intended to represent a universally validated cutoff, but rather to facilitate clinical interpretation within the present cohort. Based on this threshold, patients were compared. Variables with a *P*-value less than 0.10 in the univariate analyses, were selected for inclusion in a multivariate logistic regression model for adjustment, using the stepwise backward selection method to minimize confounding effects. The magnitude of the association between CONUT category or others and SSI was expressed as odds ratios (ORs) with 95% CIs. The goodness-of-fit of the final multivariate model was evaluated using the Hosmer-Lemeshow test, with a *P*-value greater than 0.05, indicating an acceptable result. The Nagelkerke R2 value was reported to describe the explanatory ability of the model, with higher values suggesting a better model performance. Statistical significance was set at *P* < 0.05. Model discrimination was assessed by receiver operating characteristic curve analysis, and the area under the curve (AUC), equivalent to the C statistic, was reported with its 95% confidence interval.

To minimize dependence on this data-driven categorization, a sensitivity analysis was performed by entering the CONUT score as a continuous variable into the multivariable logistic regression model, with adjustment for the same covariates included in the primary analysis.

Conventional statistical analyses were performed using SPSS 27.0, and RCS analysis was conducted using R software version 4.3.2 (R Foundation for Statistical Computing, Vienna, Austria) with the rms package.

## Results

### General characteristics

During the study period, 2573 patients were surgically treated and 1,159 were excluded for various reasons, leaving 1,414 eligible patients for analysis. Among them, there were 671 (47.5%) male and 743 (52.5%) female patients, with an average age of 51.8 (SD, 13.3) years of age. Over two-thirds (972/1414) of the operations were performed by chief or deputy chief physicians; the average preoperative waiting stay was 3.2 (SD, 2.6), and the average number of operated levels was 2.3 (SD, 1.2). Within 12 months postoperatively, 43 patients developed SSI, corresponding to a cumulative incidence of 3.0%, including 16 (1.1%) deep cases and 27 (1.9%) superficial cases. Positive wound cultures were obtained in 36 SSI cases, monomicrobial infections (*n* = 34) were the overwhelming cause, and only 2 cases involved polymicrobial infection. The predominant causative pathogens were *Staphylococcus aureus (n* *=* *11), Escherichia coli (n* *=* *9)*, *Staphylococcus epidermidis* (*n* = 6), and *Pseudomonas aeruginosa* (*n* = 3).

The average value of serum albumin concentration, preoperative peripheral blood lymphocyte count and TC concentration was 4.1 (SD, 0.5) g/dL, 1,807.0 (SD, 719.2)/mm^3^, and 181.7 (SD, 42.4) mg/dL. The derived average CONUT score was 1.6 (SD, 1.8), and according to the degree of undernutrition, 840 (59.4%) patients were classified as normal, 469 (33.2%) mild, 97 (6.9%) moderate, and 8 (0.6%) as severe malnutrition.

## RCS, univariate and multivariate analyses

The RCS analysis showed a nonlinear association between CONUT score and SSI risk, with the risk beginning to rise at approximately a CONUT score of 3 (Figure 1). Accordingly, CONUT was dichotomized as <3 and ≥3 for subsequent multivariable logistic regression analyses.

**Figure 1:**
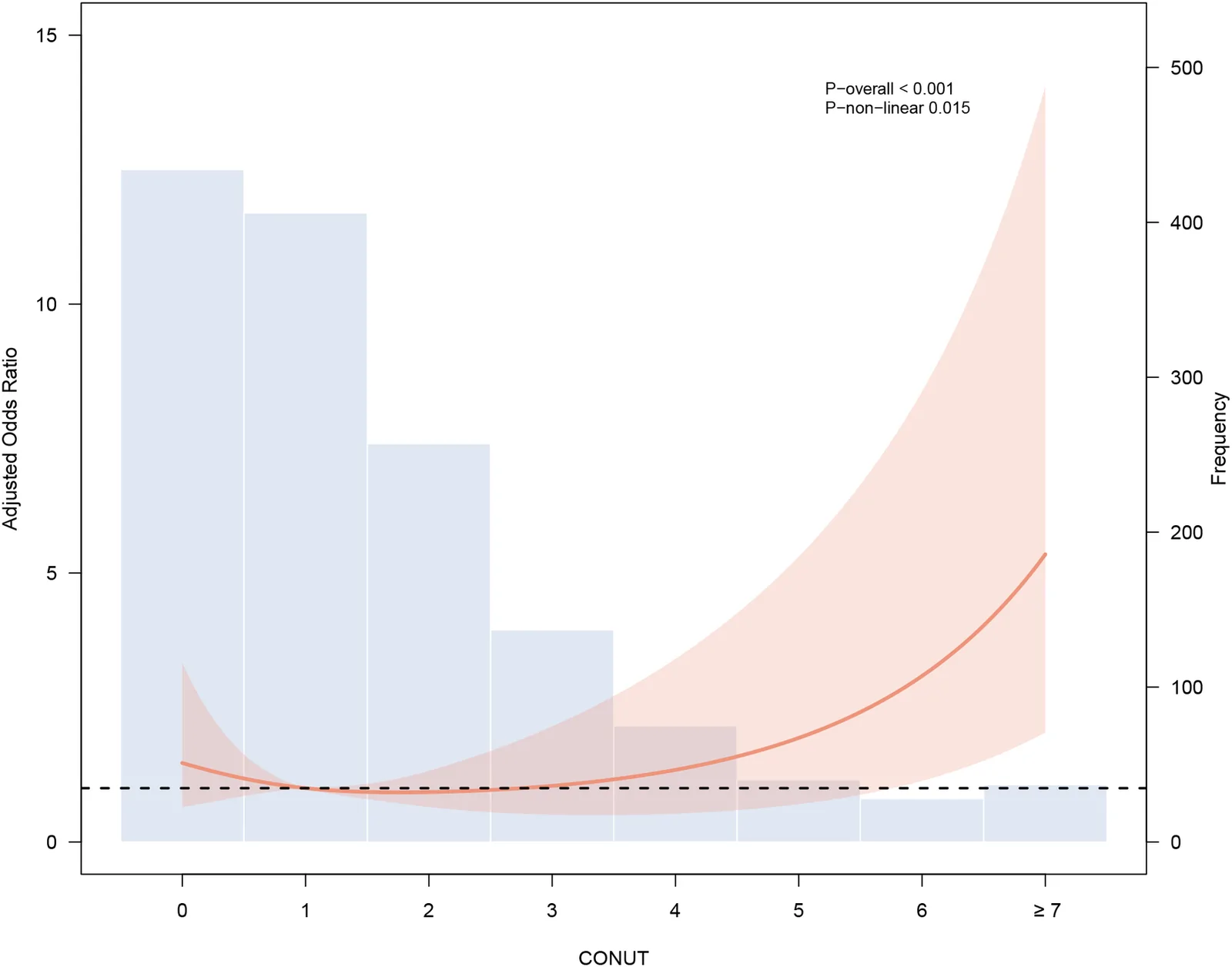
RCS analysis showing a significant nonlinear association between CONUT score and SSI risk (*P* for overall < 0.001; *P* for nonlinearity = 0.015). The solid line represents the estimated OR for SSI according to CONUT score, with a CONUT score of 0 used as the reference. The shaded area indicates the 95% CI. The histogram shows the distribution of CONUT scores in the study population. CONUT, controlling nutritional status; SSI, surgical site infection; OR, odds ratio; CI, confidence interval.

Compared with patients with CONUT scores <3 (*n* = 1,097), those with a CONUT score ≥ 3 (*n* = 317) were older and had lower BMI, higher prevalence of diabetes mellitus, cerebrovascular disease, and PVD, longer preoperative waiting times (3.7 vs. 3.1 days, *P* = 0.036), higher rates of allogeneic blood transfusion, longer surgical duration, and more frequent use of higher-generation cephalosporins. Additionally, the poor nutrition group tended to reside in rural areas (71.9% vs. 66.3%, *P* = 0.058) and had a higher rate of liver disease (4.7% vs. 2.7%, *P* = 0.074). The incidence of SSI was significantly higher in patients with CONUT scores ≥3 than in those with CONUT scores <3 (6.3% vs. 2.1%, *P* < 0.001) (Table 1**)**.

**Table 1:** Comparisons in baseline characteristics between patients with CONUT score < 3.0 and those ≥ 3.0.

Variables	Patients with CONUT score < 3.0 (*n* = 1,097)	Patients with CONUT score ≥ 3.0 (*n* = 317)	*P* value
Gender (male)	528 (48.1)	143 (45.1)	0.343
Age (years)			< 0.001
18–44	317 (28.9)	65 (20.5)	
45–64	624 (56.9)	160 (50.5)	
≥ 65	156 (14.2)	92 (29.0)	
Residence (rural place)	727 (66.3)	228 (71.9)	0.058
BMI (kg/m^2^)			0.011
< 18.5	6 (0.5)	8 (2.5)	
18.5–23.9	334 (30.4)	96 (30.3)	
24–27.9	464 (42.3)	140 (44.2)	
≥ 28.0	293 (26.7)	73 (23.0)	
Hypertension	332 (30.3)	106 (33.4)	0.282
Diabetes mellitus	99 (9.0)	51 (16.1)	< 0.001
Heart disease	154 (14.0)	51 (16.1)	0.361
Cerebrovascular disease	85 (7.7)	37 (11.7)	0.028
COPD	79 (7.2)	28 (8.8)	0.333
Renal disease	16 (1.5)	6 (1.9)	0.582
Liver disease	30 (2.7)	15 (4.7)	0.074
PVD	32 (2.9)	19 (6.0)	0.010
Recent glucocorticoid use	12 (1.1)	6 (1.9)	0.264
Preoperative wait (days)	3.1 ± 2.8	3.7 ± 3.2	0.036
Smoking status			0.529
Never	854 (77.8)	243 (76.7)	
Former	61 (5.6)	23 (7.3)	
Current	182 (16.6)	51 (16.1)	
Number of operated levels	2.3 ± 1.3	2.2 ± 1.3	0.856
Allogeneic bone or bone substitute (yes)	157 (14.3)	52 (16.4)	0.355
ASA classification			0.308
I	99 (9.0)	20 (6.3)	
II	723 (65.9)	215 (67.8)	
III or IV	275 (25.1)	82 (25.9)	
Intraoperative bleeding (mL)	549.1 ± 437.1	673.7 ± 451.2	< 0.001
Allogenic blood transfusion (yes)	229 (20.9)	91 (28.7)	0.003
Surgical time (mins)	163.0 ± 54.5	177.3 ± 56.9	0.001
Type of preoperative antibiotic use			0.009
Generation-1 cephalosporin	1,027 (93.6)	285 (89.9)	
Generation-2 cephalosporin	11 (1.0)	7 (2.2)	
Generation-3 cephalosporin	14 (1.3)	12 (3.8)	
Others	45 (4.1)	13 (4.1)	
SSI	23 (2.1)	20 (6.3)	< 0.001

CONUT, controlling nutritional status; BMI, body mass index; COPD, chronic obstructive pulmonary disease; PVD, peripheral vascular disease; ASA, American Society of Anesthesiologists; SSI, surgical site infection.

Table 2 summarizes the results of the multivariate analysis. After adjustment for potential confounders or covariates, a CONUT score ≥3 was associated with a 2.49-times increased risk of SSI, as well as comorbid diabetes mellitus (OR, 2.52; *P* = 0.014), intraoperative blood loss (OR, 1.13 per 100-mL increase, *P* < 0.001) and preoperative waiting (OR, 1.14 per 1-day increase, *P* < 0.001); allogeneic blood transfusion showed a nonsignificant trend toward increased SSI risk (OR, 2.27; *P* = 0.065). The H-L test showed an acceptable goodness-of-fit (X^2^ = 10.778, *P* = 0.215; Nagelkerke R^2^ = 0.135). The final multivariable model yielded an AUC/C statistic of 0.817 (95% CI, 0.759 to 0.875), indicating good discriminative ability for SSI (Figure 2).

**Table 2:** Multivariate analysis of CONUT in association with SSI after instrumented PLIF and adjusting for a range of covariates or confounders.

Variables	β	Standard error	OR (95% CI)	*P*
CONUT (≥3 vs. < 3)	0.912	0.324	2.49 (1.32 to 4.70)	0.005
Diabetes mellitus	0.923	0.375	2.52 (1.21 to 5.25)	0.014
Intraoperative blood bloss (for each 100 mL)	0.118	0.026	1.13 (1.07 to 1.18)	< 0.001
Allogenic blood transfusion	0.822	0.446	2.27 (0.95 to 5.43)	0.065
Preoperative waiting (for each day increment)	0.127	0.033	1.14 (1.06 to 1.21)	< 0.001

CONUT, controlling nutritional status; OR, odds ratio; CI, confidence interval.

β, indicating regression coefficient.

**Figure 2:**
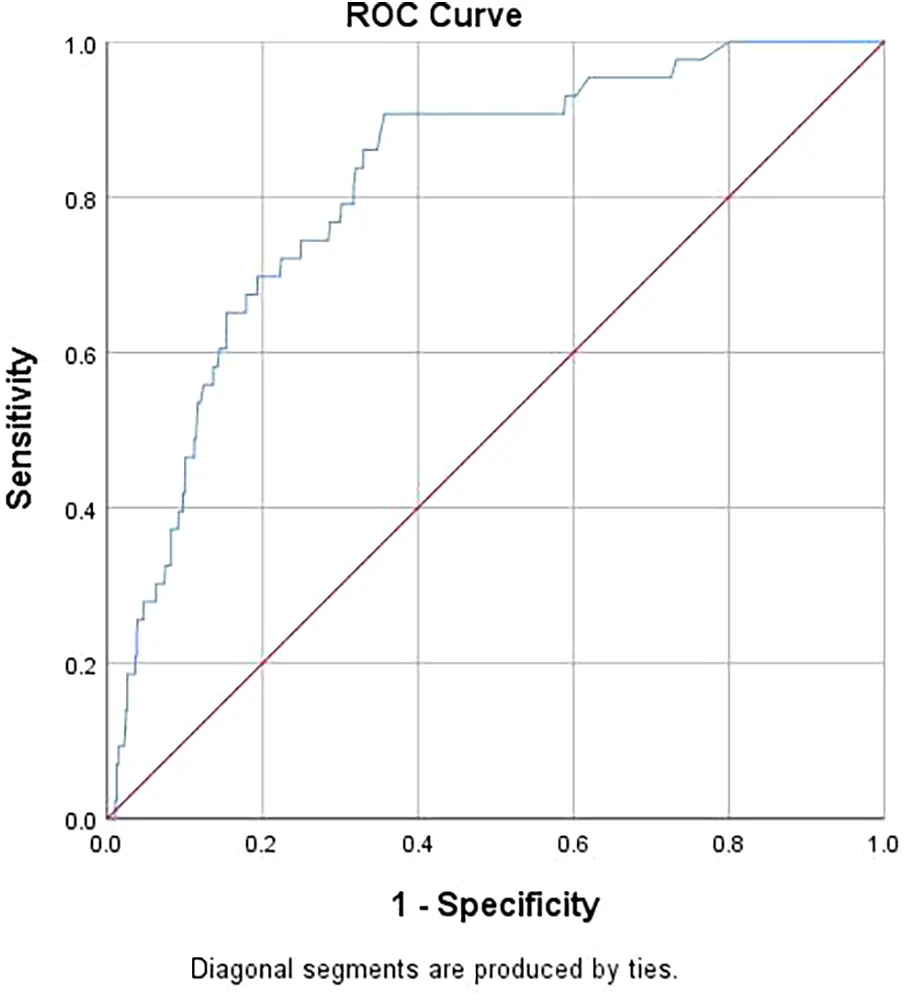
Receiver operating characteristic curve of the final multivariable logistic regression model for predicting SSI after instrumented PLIF. The model yielded an AUC/C statistic of 0.817 (95% CI, 0.759 to 0.875). AUC, area under the curve; CI, confidence interval; PLIF, posterior lumbar interbody fusion; ROC, receiver operating characteristic; SSI, surgical site infection.

In the sensitivity analysis, when the CONUT score was entered into the multivariable logistic regression model as a continuous variable, each 1-point increase in CONUT score was independently associated with a higher risk of SSI (OR, 1.28; 95% CI, 1.12–1.45; *P* < 0.001), further supporting the robustness of the primary findings. The model showed acceptable goodness-of-fit (Hosmer–Lemeshow test: X^2^ = 7.184, *P* = 0.517; Nagelkerke R^2^ = 0.100).

## Discussion

In this retrospective cohort of 1,414 patients undergoing instrumented PLIF, a higher preoperative CONUT score was independently associated with increased SSI risk. RCS analysis further suggested a nonlinear risk pattern, with SSI risk beginning to rise at approximately a CONUT score of 3. Consistently, patients with CONUT scores ≥3 had a 2.49-fold higher risk of SSI after adjustment for potential confounders, and this association remained robust when CONUT was analyzed as a continuous variable.

The observed association between CONUT score and SSI risk may be explained by the multidimensional nature of this index. Previous studies have shown that higher CONUT scores are associated with adverse postoperative outcomes in orthopaedic patients, including mortality, delayed recovery, loss of independent ambulation, and impaired functional restoration (19–21, 23). CONUT has also been applied in other clinical fields, such as oncology and internal medicine, as a marker of nutritional and physiological vulnerability (24, 25). These findings suggest that CONUT may capture a broader risk profile than nutritional status alone, incorporating aspects of host reserve, immune competence, and systemic inflammatory burden that are relevant to postoperative infection.

The three components of CONUT may each contribute to the biological plausibility of its association with SSI. Serum albumin reflects not only nutritional reserve but also inflammatory status and hepatic synthetic function; reduced albumin levels may be associated with impaired wound healing and reduced physiological resilience (26). Total cholesterol may partly reflect metabolic and nutritional reserve, while also being involved in cellular membrane integrity and immune signaling (27). Lymphocyte count is closely related to cellular immune function, and lymphopenia may indicate impaired host defense against postoperative infection (28). Therefore, by integrating albumin, total cholesterol, and lymphocyte count, the CONUT score may provide a more comprehensive assessment of preoperative nutritional, metabolic, and immune-inflammatory status than any single biomarker. This may explain why elevated CONUT scores were associated with increased SSI risk after instrumented PLIF in the present study.

In addition, the pathogen profile in our cohort provides further context for the observed association between CONUT score and SSI. Most culture-positive infections were monomicrobial, with Staphylococcus aureus, Escherichia coli, Staphylococcus epidermidis, and Pseudomonas aeruginosa being the most common pathogens. This spectrum suggests that postoperative SSI after instrumented PLIF may involve both skin-derived and Gram-negative organisms, underscoring the importance of host nutritional and immune status in infection susceptibility. Because of the limited number of culture-positive SSI cases, pathogen-specific analyses were not performed and warrant further investigation.

In this study, RCS analysis suggested that the risk of SSI began to increase when the CONUT score approached 3. Therefore, a CONUT score of 3 was selected as a clinically interpretable threshold for subsequent categorical analyses, rather than relying solely on the traditional CONUT classification of nutritional status. Similar thresholds have been used in previous studies across different clinical settings (29, 30), supporting the potential clinical relevance of mild nutritional impairment as reflected by CONUT score. However, this cutoff should be regarded as exploratory and cohort-specific, and further external validation is required before it can be applied as a universal threshold for SSI risk stratification after instrumented PLIF.

The magnitude of the association between CONUT ≥3 and SSI risk was comparable to that observed for diabetes mellitus, a well-established risk factor for postoperative infection. The clinical relevance of this finding is further supported by growing evidence that perioperative nutritional optimization may improve postoperative recovery in orthopaedic and spine surgery (9–12). In this context, CONUT may not only serve as a marker of SSI risk, but also provide a practical entry point for identifying patients who may benefit from more detailed nutritional assessment and individualized perioperative optimization. Nevertheless, whether CONUT-guided nutritional intervention can specifically reduce SSI after instrumented PLIF remains to be confirmed in future prospective studies.

This study has several strengths, including a relatively large overall cohort and a comprehensive, multi-source approach for identifying SSI cases through medical records, readmissions, follow-up visits, and telephone interviews. However, several limitations should be acknowledged. First, the retrospective design inevitably introduced the possibility of information bias, and some comorbidity data may have relied partly on patient self-report or incomplete medical documentation. Second, although SSI events were ascertained using multiple sources, superficial infections may still have been underreported, particularly after discharge, which could have led to an underestimation of the true SSI incidence. Third, although same-day preoperative laboratory results were preferentially used for CONUT calculation, the three components were not necessarily measured on the same day or derived from the same specimen tube in all patients, which may have introduced measurement variability. Fourth, despite multivariable adjustment, residual confounding from unmeasured or inadequately captured factors cannot be excluded. Fifth, although the overall sample size was relatively large, the number of SSI events was limited, which may affect the stability of multivariable estimates and the precision of risk estimates in patients with high CONUT scores. Finally, this was a single-center study conducted at a high-volume tertiary institution; therefore, the generalizability of the findings, including the clinically interpretable CONUT threshold of 3, requires further validation in external cohorts.

In summary, a higher preoperative CONUT score was independently associated with increased SSI risk after instrumented PLIF, with RCS analysis suggesting a risk increase beginning at approximately a CONUT score of 3. Given its accessibility and low cost, CONUT may serve as a practical indicator for preoperative nutritional assessment and SSI risk stratification, although further external validation is warranted.

## Data Availability

The raw data supporting the conclusions of this article will be made available by the authors, without undue reservation.
