# Supplementary material for: Association between preoperative controlling nutritional status score and surgical site infection following instrumented posterior lumbar interbody fusion
Source: Front Surg. 2026 Sep 24;13:1918332. doi: 10.3389/fsurg.2026.1918332 (PMC13645754; doi:10.3389/fsurg.2026.1918332)
Supplement: Supplementary file 1 [file Table1.docx]

**Supplementary Table 1** Scoring system for the Controlling Nutritional Status (CONUT)

| Degree of  undernutrition | CONUT score | Serum albumin (g/dl) | Total lymphocyte count (/mm^3^) | TC (mg/dl) |
| --- | --- | --- | --- | --- |
| Normal | 0-1 | ≥ 3.5 (0) | ≥1600 (0) | ≥180 (0) |
| Mild | 2-4 | 3.00-3.49 (2) | 1200-1599 (1) | 140-179 (1) |
| Moderate | 5-8 | 2.50-2.99 (4) | 800-1199 (2) | 100-139 (2) |
| Severe | 9-12 | < 2.50 (6) | <800 (3) | <100 (3) |

CONUT score = serum albumin score + total lymphocyte score + total cholesterol score.
